# Effects of Nutraceuticals on Cisplatin-Induced Cytotoxicity in HEI-OC1 Cells

**DOI:** 10.3390/ijms242417416

**Published:** 2023-12-12

**Authors:** Lorenzo Guidotti, Elena Tomassi, Silvia Marracci, Michele Lai, Dominga Lapi, Rossana Pesi, Laura Pucci, Ettore Novellino, Elisabetta Albi, Mercedes Garcia-Gil

**Affiliations:** 1 General Physiology Unit, Department of Biology, University of Pisa, Via San Zeno 31, 56127 Pisa, Italy; l.guidotti1@student.unisi.it (L.G.); silvia.marracci@unipi.it (S.M.); dominga.lapi@unipi.it (D.L.); 2 Institute of Agricultural Biology and Biotechnology, Italian National Research Council, Via Moruzzi 1, 56124 Pisa, Italy; elena.tomassi@ibba.cnr.it (E.T.); pucci@ibba.cnr.it (L.P.); 3 Retrovirus Centre, Department of Translational Medicine and New Technologies in Medicine and Surgery, University of Pisa, Strada Statale del Brennero 2, 56127 Pisa, Italy; michele.lai@unipi.it; 4 Biochemistry Unit, Department of Biology, University of Pisa, Via San Zeno 31, 56127 Pisa, Italy; rossana.pesi@unipi.it; 5 Facoltà di Medicina e Chirurgia, Università Cattolica del Sacro Cuore, Largo Francesco Vito 1, 00168 Rome, Italy; ettore.novellino@unicatt.it; 6 Department of Pharmaceutical Sciences, Interno Orto Botanico, University of Perugia, Via Romana, 06126 Perugia, Italy; elisabetta.albi@unipg.it; 7 Interdepartmental Research Center Nutrafood “Nutraceuticals and Food for Health”, University of Pisa, 56124 Pisa, Italy

**Keywords:** cisplatin, ototoxicity, Lisosan G, Taurisolo^®^, erucin, hydrogen disulfide donor, acid sphingomyelinase, HEI-OC1 cell line

## Abstract

Cisplatin is a chemotherapeutic drug for the treatment of several solid tumors, whose use is limited by its nephrotoxicity, neurotoxicity, ototoxicity, and development of resistance. The toxicity is caused by DNA cross-linking, increase in reactive oxygen species and/or depletion of cell antioxidant defenses. The aim of the work was to study the effect of antioxidant compounds (Lisosan G, Taurisolo^®^) or hydrogen sulfide (H_2_S)-releasing compounds (erucin) in the auditory HEI-OC1 cell line treated with cisplatin. Cell viability was determined using the MTT assay. Caspase and sphingomyelinase activities were measured by fluorometric and colorimetric methods, respectively. Expression of transcription factors, apoptosis hallmarks and genes codifying for antioxidant response proteins were measured by Western blot and/or RT-qPCR. Lisosan G, Taurisolo^®^ and erucin did not show protective effects. Sodium hydrosulfide (NaHS), a donor of H_2_S, increased the viability of cisplatin-treated cells and the transcription of heme oxygenase 1, superoxide dismutase 2, NAD(P)H quinone dehydrogenase type 1 and the catalytic subunit of glutamate-cysteine ligase and decreased reactive oxygen species (ROS), the Bax/Bcl2 ratio, caspase-3, caspase-8 and acid sphingomyelinase activity. Therefore, NaHS might counteract the cytotoxic effect of cisplatin by increasing the antioxidant response and by reducing ROS levels and caspase and acid sphingomyelinase activity.

## 1. Introduction

Cisplatin is a cytotoxic drug widely used for the treatment of several types of solid tumors, including head and neck, lung, bladder, ovarian, and testicular cancers [1]. The efficiency of cisplatin is limited by its nephrotoxicity, neurotoxicity, ototoxicity and tumor resistance [1,2,3,4,5]. Hearing loss after ototoxic damage is permanent and affects language and social development especially for young children [6].

After entering cells, cisplatin undergoes hydration and loss of chlorine ions, leading to the cross-linking of nuclear and mitochondrial DNA [4,7], increasing reactive oxygen species (ROS), and/or depletion of cellular antioxidant defenses [8], resulting in cell death due to the inhibition of DNA replication and cellular metabolism [9]. Accumulating evidence indicates the involvement of inflammation, endoplasmic reticulum stress, autophagy, necroptosis, and intrinsic apoptosis in cisplatin-induced cell death [10].

Different preclinical pharmacological strategies have been proposed to reduce the ototoxic effects of cisplatin, although none has been approved for clinical practice [10]. It has been suggested that the administration of antioxidant agents such as N-acetyl cysteine, sodium thiosulfate, lipoic acid and polyphenolic species can alleviate cisplatin toxic side-effects [1,11,12,13]. In addition, bismuth compounds also reduce cisplatin-induced nephrotoxicity by enhancing glutathione conjugation and cisplatin sequestration to vesicles [14]. Accumulating evidence indicates that H_2_S has neuroprotective effects [15,16,17]. Moreover, clinical trials have shown that administration after cisplatin chemotherapy of sodium thiosulfate, considered to be a H_2_S donor, was not associated with adverse events and resulted in a lower incidence of hearing loss [18,19].

The aim of the present work was to study whether different nutrients with antioxidant or H_2_S donor properties might protect from cisplatin-induced cytotoxicity using as the murine cell line House Ear Institute-Organ of Corti 1 (HEI-OC1). This cell line expresses specific markers of cochlear hair cells and supporting cells [20] and it is the most widely used cell model for ototoxicity studies. In particular, we have used Lisosan G, a fermented power obtained from whole grains, (*Triticum aestivum*), [21], a novel nutraceutical formulation based on grape polyphenols (registered as Taurisolo^®^, MB-Med, Turin, Italy) [22,23,24], and erucin, an H_2_S-donor [25,26]. Lisosan G has been registered at the Italian Ministry of Health as a nutritional supplement. It is enriched in bioactive substances such as flavonoids and flavonols, tocopherols, polyunsaturated fatty acids and alpha-lipoic acid [21,27,28,29]. Both in vitro and in vivo studies have shown that Lisosan G has protective effects on different cell types and tissues including hepatocytes, microvascular endothelial cells and the retina through the control of both oxidative and inflammatory processes [27,28,29,30,31]. Taurisolo^®^ has been demonstrated to have antioxidant activity in neutrophils [24], to protect rat brains from ischemia-reperfusion injuries [22], and to counteract high-glucose- and trimethylamine N-oxide-induced cytotoxicity in cardiomyoblasts [23]. Erucin is widely present in edible plants belonging to the *Brassicaceae* family and significantly protect against, in human endothelial and vascular smooth muscle cells, the increase in ROS, caspase 3/7 activation, and tumor necrosis factor α (TNFα) and interleukin 6 (IL-6) levels associated with high glucose concentrations [26]. None of these nutraceuticals was able to have a protective effect on cisplatin-induced cytotoxicity. However, the H_2_S-donor NaHS increased viability in cisplatin-treated HEI-OC1 cells. In cisplatin-treated cells, NaHS decreased the level of ROS, the ratio of Bax/Bcl2 expression, the activity of caspase-3, caspase-9 and acid sphingomyelinase (aSMase) and increased the transcription of the genes coding for heme oxygenase 1 (*Hmox1*), superoxide dismutase 2 (*Sod2*), NADPH quinone dehydrogenase type 1 (*Nqo1*) and glutamate-cysteine ligase catalytic subunit (*Gclc*).

## 2. Results

### 2.1. Effect of Cisplatin, Lisosan G, Taurisolo^®^, Erucin, and NaHS on Viability of HEI-OC1 Cells

**Figure 1:**
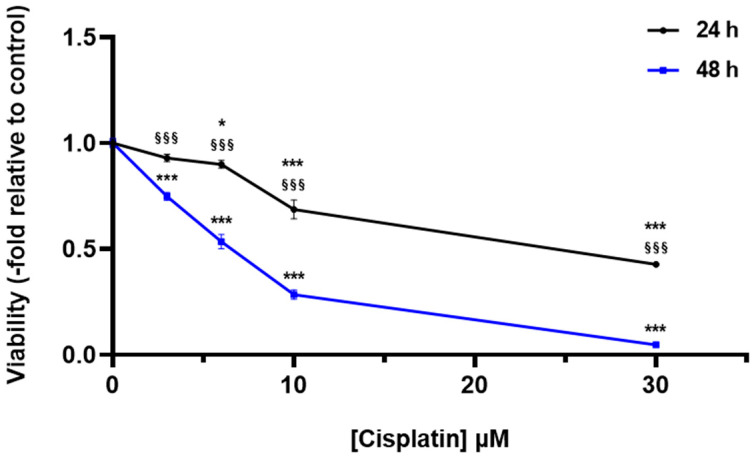
Effect of cisplatin incubation time and dose on the viability of HEI-OC1 cells. HEI-OC1 cells were incubated for 24 or 48 h with different concentrations of cisplatin, and viability was measured as described in Section 4. Data were normalized to the absorbance of cells incubated in the absence of cisplatin (control) and are expressed as the mean ± SEM (*n* = 6). Significance: * *p* < 0.05; *** *p* < 0.001 vs. control; ^§§§^ *p* < 0.001 vs. 48 h.

**Figure 2:**
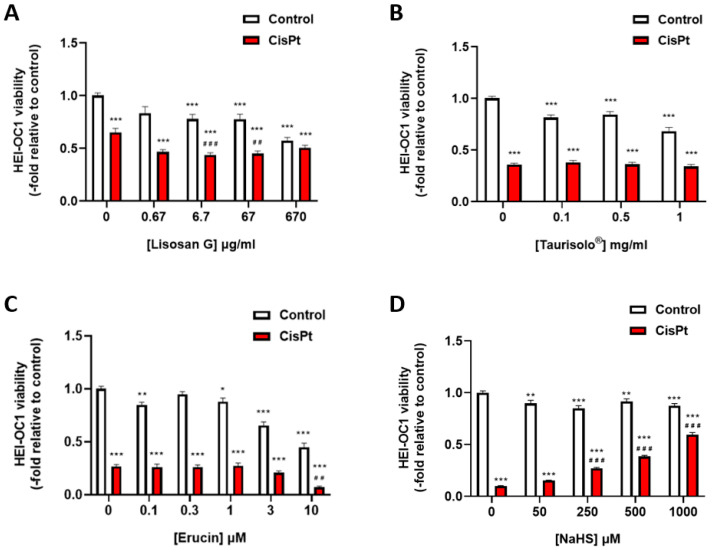
Effect of Lisosan G, Taurisolo^®^, erucin and NaHS on the viability of HEI-OC1 cells. (**A**) HEI-OC1 cells were preincubated for 4 h with different concentrations of Lisosan G and then treated for 24 h in the presence or absence of 10 μM cisplatin, and viability was measured as described in Section 4 (*n* = 2); (**B**) cells were preincubated for 1 h with different concentration of Taurisolo^®^ and then incubated with or without 5 μM cisplatin for 48 h (*n* = 4). (**C**) cells were preincubated for 1 h with different concentrations of erucin and then in the presence or absence of 5 μM cisplatin for 48 h (*n* = 4). (**D**) cells were incubated in the presence or the absence of different concentrations of NaHS and 10 μM cisplatin for 48 h (*n* = 3). Data were normalized to control cells and are expressed as the mean ± SEM. Significance: * *p* < 0.05; ** *p* < 0.01; *** *p* < 0.001; vs. control; ^##^ *p* < 0.01; ^###^ *p* < 0.001 vs. cisplatin. CisPt, cisplatin; NaHS, sodium hydrosulfide.

Cisplatin is cytotoxic for HEI-OC1 cells in dose-, time- and cell density-dependent manners (Figure 1 and Figure S1). We have tested whether the different antioxidant compounds or H_2_S donors were able to reduce cisplatin-induced cytotoxicity. Lisosan G decreased viability in a dose-dependent manner both in control cells and in cells treated with cisplatin (Figure 2A). Taurisolo^®^ lightly reduced the viability of control cells without modifying viability in cisplatin-treated cells (Figure 2B). Erucin > 3 mM was toxic for control cells, and it was not able to increase viability in cisplatin-treated cells (Figure 2C). However, NaHS, an H_2_S donor, was able to increase viability in cisplatin-treated cells in a dose-dependent manner (Figure 2D).

**Figure 3:**
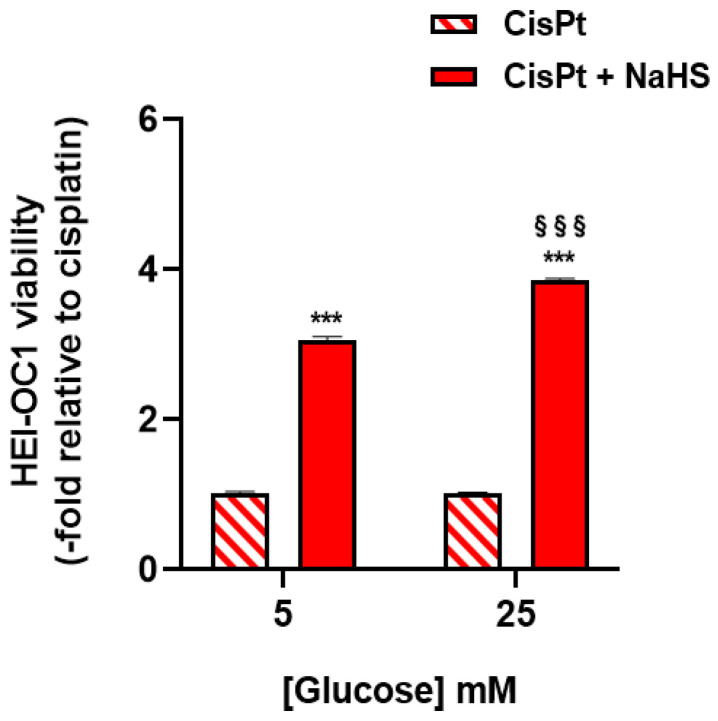
Effect of glucose on the viability of HEI-OC1 treated with 10 μM cisplatin and 500 μM NaHS for 48 h. Cells were incubated in the presence or the absence of different concentrations of glucose (*n* = 2, performed in octuplicate). Data were normalized to cisplatin-treated cells in the absence of NaHS in each condition and are expressed as the mean ± SEM. Significance: *** *p* < 0.001 vs. cisplatin; ^§§§^ *p* < 0.001 vs. 5 mM of glucose.

Since the survival effect of NaHS could be glucose-dependent [32], viability was also measured in the presence of a lower glucose concentration (5 mM of glucose instead of 25 mM of glucose). The effect of NaHS was reduced at the lower glucose concentration (Figure 3 and Figure S2).

**Figure 4:**
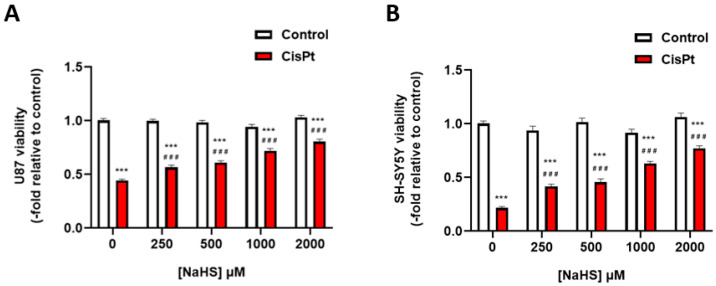
Effect of NaHS on the viability of U87 human glioblastoma cells and SH-SY5Y human neuroblastoma cells. (**A**) U87 cells were incubated in the presence or the absence of different concentrations of NaHS and 10 μM cisplatin for 48 h (*n* = 3). (**B**) SH-SY5Y neuroblastoma cells were incubated in the presence or the absence of different concentrations of NaHS and 10 μM cisplatin for 24 h (*n* = 3). Data were normalized to control cells and are expressed as the mean ± SEM. Significance: *** *p* < 0.001; vs. control; ^###^ *p* < 0.001 vs. cisplatin. CisPt, cisplatin; NaHS, sodium hydrosulfide.

In order to investigate the specificity of the action of NaHS on cisplatin-induced cytotoxicity, the viability assay was also performed in U87 human glioblastoma cells and SH-SY5Y human neuroblastoma cells (Figure 4). NaHS had a dose-dependent protective effect on cisplatin-induced cytotoxicity.

### 2.2. NaHS Significantly Reduced Cisplatin-Induced Apoptosis in HEI-OC1 Cells

**Figure 5:**
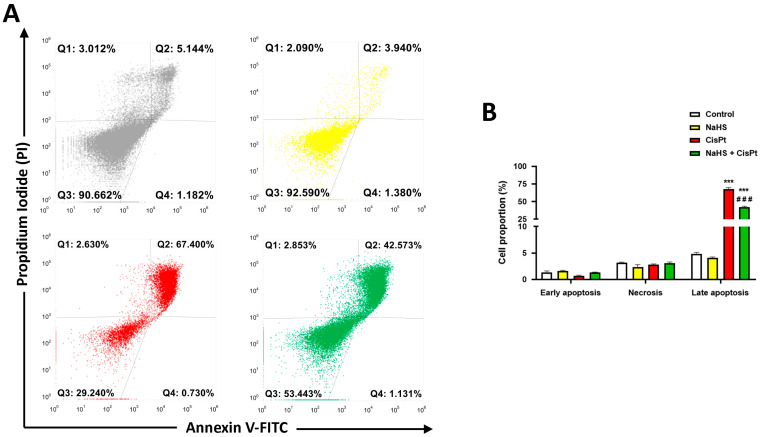
Flow cytometry analysis. HEI-OC1 cells were incubated in the presence or the absence of 10 μM cisplatin and 500 μM NaHS for 48 h (*n* = 3) and the type of cell death was analyzed by flow cytometry. (**A**) Representative data of flow cytometry. Late apoptosis (right-upper panel), early apoptosis (right-bottom panel) and necrosis (left-upper panel) were quantified in (**B**). Data are expressed as the mean ± SEM. Significance: *** *p* < 0.001; vs. control; ^###^ *p* < 0.001 vs. cisplatin. CisPt, cisplatin; NaHS, sodium hydrosulfide.

Flow cytometry analysis showed that the decrease in viability induced by 10 μM cisplatin for 48 h was mainly due to apoptosis (Figure 5A,B). Apoptotic cells which totaled 4.9% in the controls, increased to 67.6% after treatment with cisplatin, and to 42.6% after the treatment with cisplatin plus 500 μM NaHS. Therefore, the combination NaHS plus cisplatin significantly reduced apoptosis compared to cisplatin-treated cells (Figure 5B).

### 2.3. Effect of Cisplatin and NaHS on the Expression and Activity of Apoptosis-Associated Proteins in HEI-OC1 Cells

**Figure 6:**
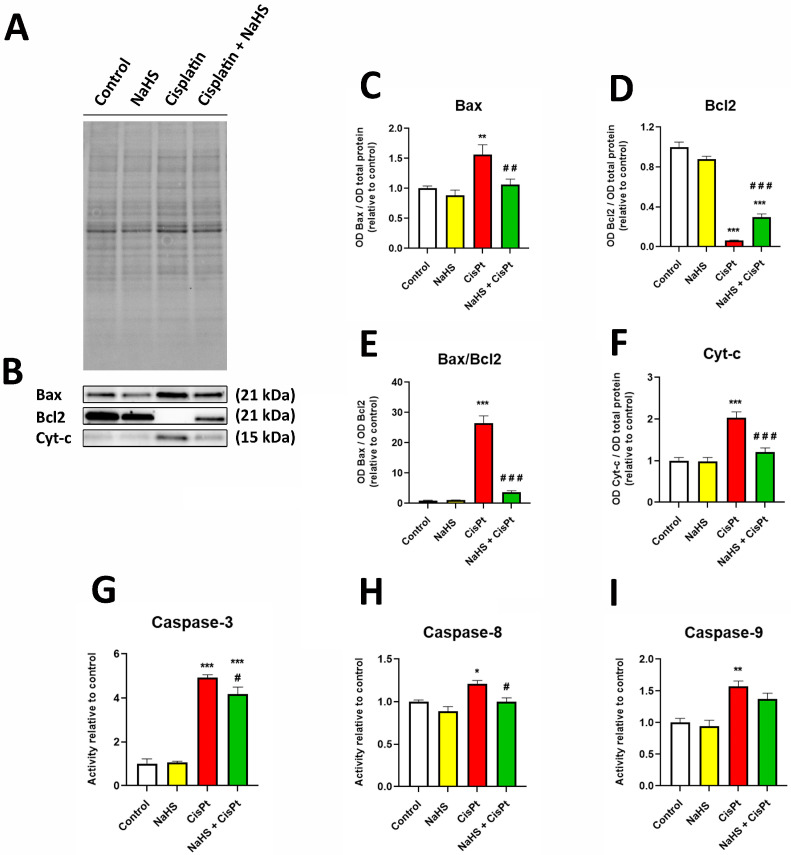
Effect of NaHS and cisplatin on the expression of Bcl2, Bax and cytochrome c and on the activity of caspases in HEI-OC1 cells. (**A**) Representative example of total transferred proteins of extracts of HEI-OC1 cells treated for 24 h in the presence or absence of 10 µM cisplatin and 500 µM NaHS (**B**) Representative example of Western blots. (**C**–**F**) Quantification of the expression of Bax, Bcl2 and the ratio Bax/Bcl2 (*n* = 6) and cytochrome c (*n* = 8). Data have been normalized to the total quantity of proteins and to the control and are expressed as the mean ± SEM. (**G**–**I**) HEI-OC1 cells were treated with or without 10 μM cisplatin and 500 μM NaHS for 48 h and the activities of (**G**) caspase-3, (**H**) caspase-8 and (**I**) caspase-9 were measured as variation of absorbance/time as described in Section 4 and normalized to the values of control cells. Data are expressed as the mean ± SEM (*n* = 4). Statistical significance: * *p* < 0.05; ** *p* < 0.01, *** *p* < 0.001 vs. control; ^#^ *p* < 0.05; ^##^ *p* < 0.01, ^###^ *p* < 0.001 vs. cisplatin. CisPt, cisplatin; Cyt-c, cytochrome c; NaHS, sodium hydrosulfide.

In another series of experiments, we studied apoptosis markers such as Bax, Bcl2, cytochrome c and caspases. The expression of Bax and cytochrome c was increased while the Bcl-2 expression was decreased in HEI-OC1 cells treated with 10 μM cisplatin for 24 h. The presence of 500 μM NaHS reversed these effects in cisplatin-treated cells (Figure 6A–F). We have also measured the activity of the initiator caspase-8 and -9 and the executioner caspase-3 in the presence and absence of 10 μM cisplatin and 500 μM NaHS for 48 h. Cisplatin significantly increased the activity of the three caspases and NaHS reduced the cisplatin-stimulated activity of caspase-3 and -8 (Figure 6G–I).

### 2.4. NaHS Significantly Reduced the Cisplatin-Induced Increase in ROS in HEI-OC1 Cells

**Figure 7:**
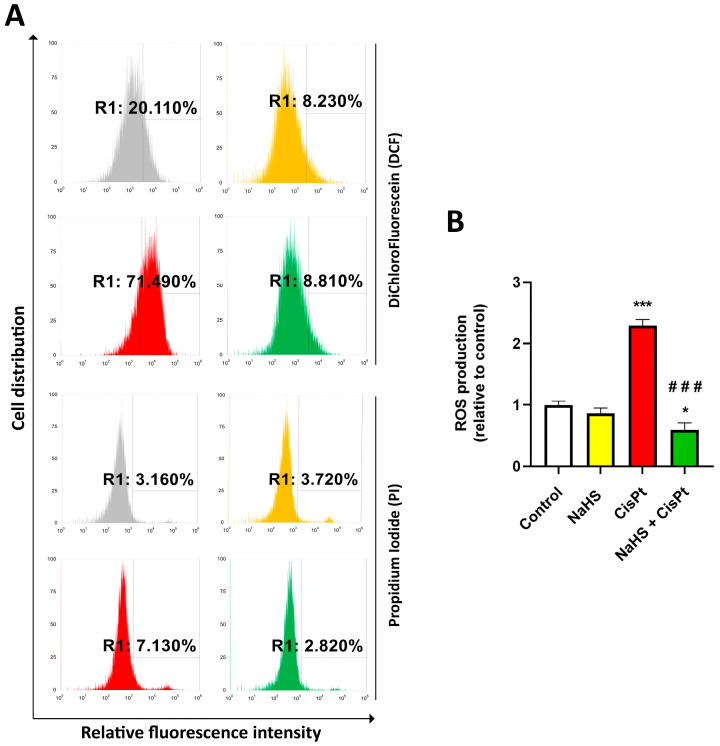
Measurement of ROS production. HEI-OC1 cells were incubated in the presence or the absence of 10 μM cisplatin and 500 µM NaHS for 24 h and ROS production was analyzed by flow cytometry. (**A**) Representative data of flow cytometry. (**B**) Quantification. Data are expressed as the mean ± SEM (*n* = 6). Statistical significance: * *p* < 0.05, *** *p* < 0.001 vs. control; ^###^ *p* < 0.001 vs. cisplatin. CisPt, cisplatin; NaHS, sodium hydrosulfide.

**Figure 8:**
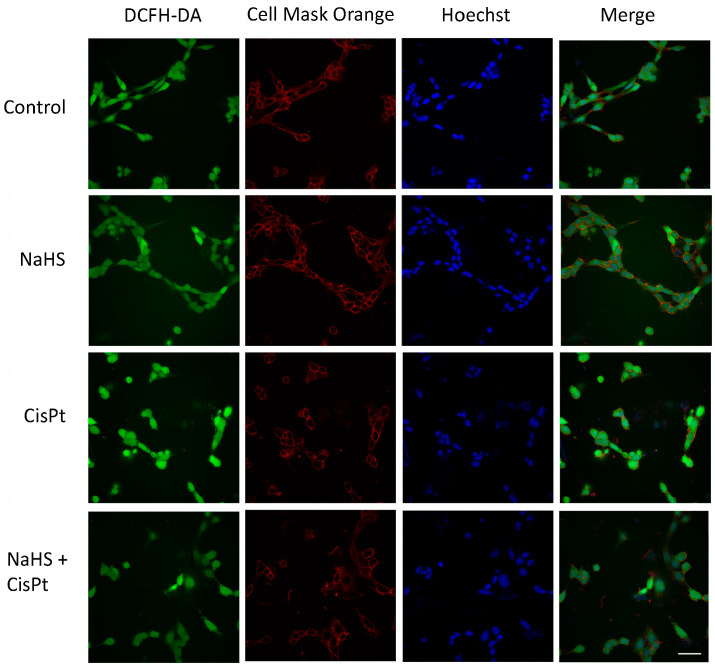
Cells were seeded in 96-well plates, treated for 24 h in the presence or absence of 10 μM cisplatin and 500 μM NaHS, incubated with 10 μM DCFDA, 1 μg/mL Cell Mask Orange and 1 μg/mL Hoechst 33342 for 30 min. The figure shows representative images acquired using the Operetta CLS high-content imaging device. Scale bar 50 μm.

To investigate the effects of NaHS on intracellular ROS generation induced by cisplatin, HEI-OC1 cells were treated with 10 µM cisplatin in the presence or absence of 500 µM NaHS for 24 h, incubated with 2′-7′-dichlorodihydrofluorescein diacetate (DCFH-DA) and ROS levels were measured by cytofluorimetry (Figure 7A). Cisplatin significantly increased ROS generation (2.29 ± 0.14-fold compared to control). ROS production in the cells treated with both NaHS and cisplatin was significantly lower (0.59 ± 0.11-fold compared to the control) than that of the cells treated with cisplatin (Figure 7B). Figure 8 shows representative images acquired using the Operetta CLS high-content imaging device.

### 2.5. Effect of Cisplatin and NaHS on Nrf2, Sod2, Hmox1, Nqo1 and Gclc Gene Expression in HEI-OC1 Cells

**Figure 9:**
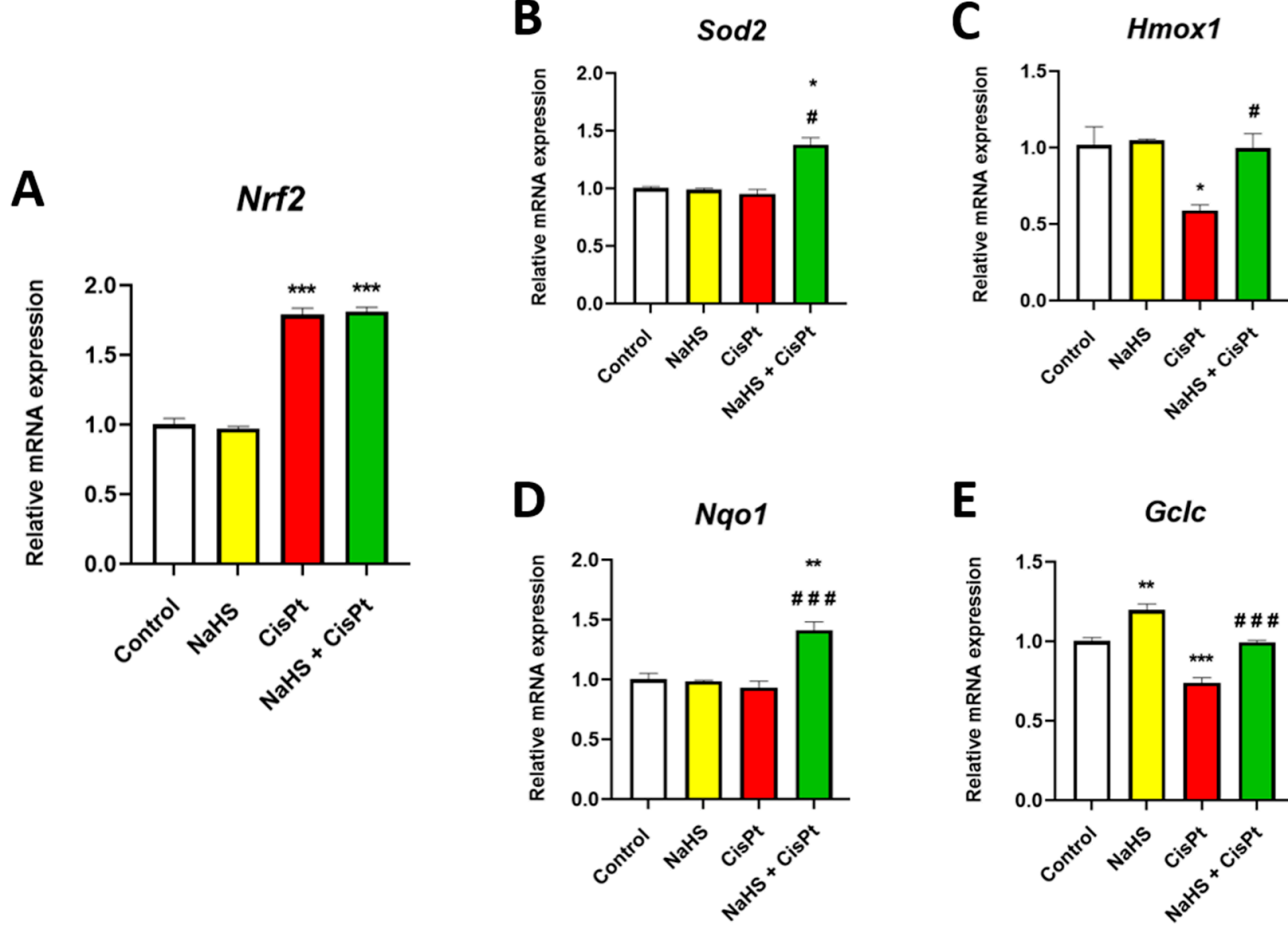
Effect of NaHS and cisplatin on *Nrf2*, *Sod2*, *Hmox1*, *Nqo1* and *Gclc* gene expression in HEI-OC1 cells. HEI-OC1 cells were treated for 24 h in the presence or absence of 10 µM cisplatin and 500 μM NaHS. The gene expression levels of *Nrf2* (**A**), *Sod2* (**B**), *Hmox 1* (**C**), *Nqo1* (**D**), and *Gclc* (**E**) were calculated as 2^−ΔΔCt^ using *Rpl13A* as an endogenous housekeeping gene and normalized to controls. Data are expressed as the mean ± SEM from *n* = 3 independent samples measured in triplicate. Statistical significance was estimated by one-way ANOVA: * *p* < 0.05; ** *p* < 0.01, *** *p* < 0.001 vs. control; ^#^ *p* < 0.05, ^###^ *p* < 0.001 vs. cisplatin. CisPt, cisplatin; *Gclc*, glutamate-cysteine ligase catalytic subunit; *Hmox1*, heme oxygenase 1 gene; NaHS, sodium hydrosulfide; *Nqo1*, NADPH quinone dehydrogenase type 1; *Nrf2*, nuclear factor erythroid 2-related factor; *Sod2*, superoxide dismutase 2 gene.

In order to study the effect of cisplatin and NaHS on the antioxidant system of HEI-OC1 cells, we studied the expression of some genes that codify for proteins playing a crucial role in the antioxidant response such as *nuclear factor erythroid 2-related factor 2* (*Nrf2*), *Hmox1*, *Sod2*, *Nqo1* and *glutamate-cysteine ligase catalytic subunit* (*Gclc*) [33,34,35,36]. We have observed that treatment with cisplatin for 24 h increases the expression of *Nrf2*, decreases the expression of *Hmox1* and *Gclc* and does not modify the expression of *Sod2* and *Nqo1* (Figure 9A–E). Incubation with NaHS significantly increases the expression of *Gclc* in control cells. Incubation in the presence of both cisplatin and NaHS significantly increases the expression of *Hmox1*, *Sod2*, and *Nqo1* and *Gclc* compared to cisplatin-treated cells, suggesting that the increase in viability in NaHS co-treated cells could be due to an increase in the expression of the genes of the antioxidant response. The protein expression changes of Nrf2 paralleled those of mRNA in response to cisplatin and NaHS (Figure S3).

### 2.6. Effect of Cisplatin and NaHS on the Expression of NFkB, pNFkB, STAT3 and pSTAT3 in HEI-OC1 Cells

**Figure 10:**
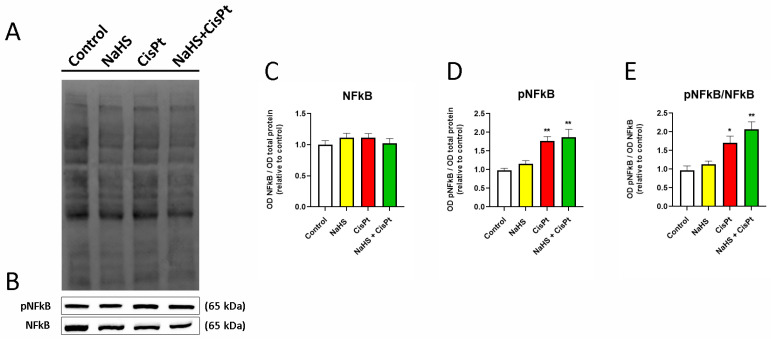
Effect of NaHS and cisplatin on NFkB and pNFkB expression in HEI-OC1 cells. HEI-OC1 cells were treated for 24 h in the presence or absence of 10 µM cisplatin and 500 μM NaHS. (**A**) Representative example of total transferred proteins. (**B**) Representative example of Western blots. (**C**–**E**) Quantification of the expression of NFkB, pNFkB and the ratio pNFkB/NFkB (*n* = 4). Data have been normalized to the total quantity of proteins and to the control and are expressed as the mean ± SEM. Statistical significance: * *p* < 0.05, ** *p* < 0.01, vs. control. CisPt, cisplatin; NaHS, sodium hydrosulfide; NFkB, nuclear factor kappa B; pNFkb, phosphorylated NFkB.

**Figure 11:**
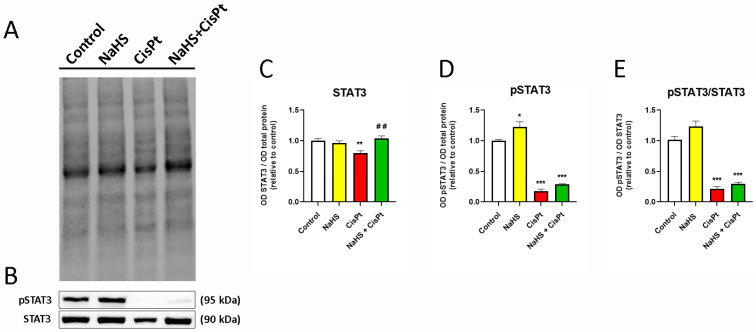
Effect of cisplatin and NaHS on STAT3 and pSTAT3 expression in HEI-OC1 cells. HEI-OC1 cells were treated for 24 h in the presence or absence of 10 µM cisplatin and 500 μM NaHS. (**A**) Representative example of total transferred proteins. (**B**) Representative example of Western blots. (**C**) Quantification of the expression of STAT3, (**D**) pSTAT3 and (**E**) the ratio pSTAT3/STAT3 (*n* = 4). Data have been normalized to the total quantity of proteins and to the control and are expressed as the mean ± SEM. Statistical significance: * *p* < 0.05, ** *p* < 0.01, *** *p* < 0.001 vs. control; ^##^ *p* < 0.01 vs. cisplatin. CisPt, cisplatin; NaHS, sodium hydrosulfide; pSTAT3, phosphorylated STAT3; STAT3, signal transducer and activator of transcription 3.

In order to clarify the mechanism underlying the protective effect of NaHS, the activation of two transcription factors involved in cisplatin-induced cytotoxicity, nuclear factor kappa B (NFkB), and signal transducer and activator of transcription 3 (STAT3) was analyzed by examining the expression of the phosphorylated (active) and unphosphorylated forms in cells treated with 500 μM NaHS and/or 10 µM cisplatin for 24 h (Figure 10 and Figure 11). There was no modification of the expression of NFkB but phosphorylated NFkB (pNFkB) increased 79% and 90%, respectively in cisplatin- and NaHS plus cisplatin-treated cells compared to control cells (Figure 10). NaHS did not significantly change the ratio of pNFkB/NFkB in cisplatin-treated cells (Figure 10E). There was no significant variation in the expression of pNFkB or NFkB after 6 h of treatment (Figure S4).

Treatment with cisplatin for 24 h decreases the expression of STAT3 compared with control cells but the presence of NaHS reverses this effect (Figure 11A–C). The active form (pSTAT3) slightly increases in NaHS-treated cells, and decreases significantly both in the presence of cisplatin alone and in the presence of the combination of NaHS and cisplatin (Figure 11A,B,D). NaHS does not modify the ratio pSTAT3/STAT3 in control or cisplatin-treated cells (Figure 11D).

### 2.7. Effect of Cisplatin and NaHS on Acid Sphingomyelinase Activity in HEI-OC1 Cells

**Figure 12:**
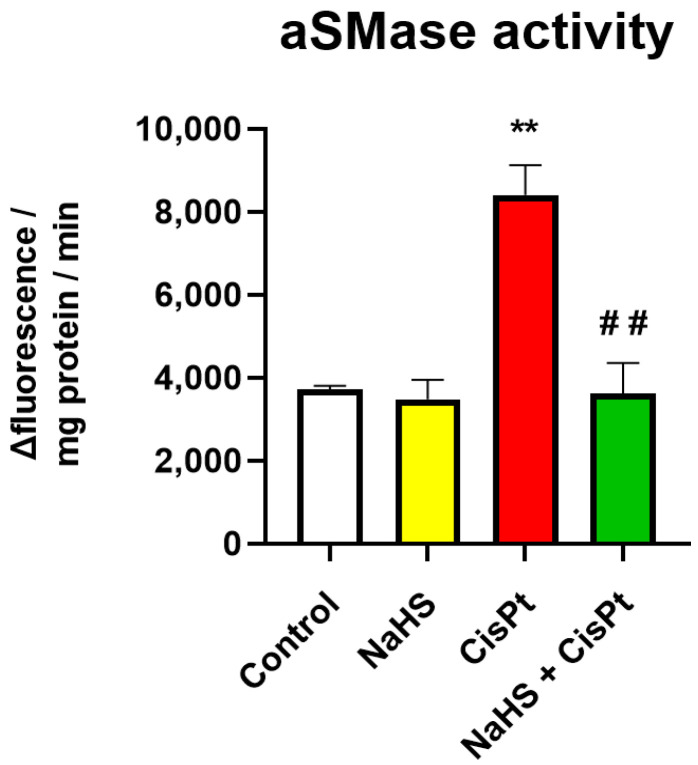
Effect of NaHS and cisplatin on aSMase activity in HEI-OC1 cells. aSMase activity was evaluated using an Amplex Red Sphingomyelinase assay kit, as reported in Section 4. Activity has been measured as variation of fluorescence/mg protein/min and is expressed as the mean ± SEM (*n* = 3, each carried out in duplicate). Significance: ** *p* < 0.01 vs. control; ^##^ *p* < 0.01 vs. cisplatin. aSMase, acid sphingomyelinase; CisPt, cisplatin, NaHS, sodium hydrosulfide.

Since cisplatin is able to activate acid aSMase and induce apoptosis in different types of cells [37,38], we measured aSMase activity in HEI-OC1 (Figure 12). We found that the activity of aSMase increases 2.2-fold in cisplatin-treated cells compared to controls and reverts to control levels in cells treated with the combination cisplatin plus NaHS.

## 3. Discussion

Cisplatin-induced damage in cochlear cells is mainly due to the production of ROS and the release of the proinflammatory molecules TNFα and IL-6 [39,40,41], leading to cellular death by apoptosis, necrosis [2], autophagy [10] and ferroptosis [42]. Much effort has been dedicated to finding substances capable of reverting ototoxicity, but at the moment none has been approved [43]. In the present work, we have tested whether compounds with antioxidant (Lisosan G, Taurisolo^®^) or H_2_S-releasing properties (erucin, NaHS) had anti-cytotoxic effects in cisplatin-treated HEI-OC1 cells. Lisosan G decreases oxidative stress-induced caspase-3 activation in explants of the retina, decreases vascular endothelial growth factor expression in the retina of diabetic rats [31] and increases the activity of antioxidant enzymes in the liver of cisplatin-treated [29] rats and primary hepatocytes [44], but it does not increase viability in cisplatin-treated HEI-OC1 cells. Taurisolo^®^ has been demonstrated to have protective effects both in vivo and in vitro [22,23]. Erucin significantly prevents the high glucose-induced decrease in cell viability as well as the increase in ROS, caspase 3/7 activation, and TNFα and IL-6 levels in human endothelial and vascular smooth muscle cells [24]. None of these compounds is effective in protecting HEI-OC1 cells from cisplatin-induced cell death. Since ototoxicity in vivo involves many types of cells, and the HEI-OC1 cells that we used are a model of the undifferentiated cells of the organ of Corti, our results do not exclude the possibility that Lisosan G, Taurisolo^®^ or other nutraceuticals could exert a protective effect in the cochlea. For example, some of these compounds could act on the blood–labyrinth barrier, whose function depends on the stria vascularis, a vascularized tissue that lines the wall of the cochlea.

Our results indicate that H_2_S donors such as NaHS are able to increase viability and decrease apoptosis measured both by cytofluorimetry and as a decrease in the expression or the activity of the hallmarks of apoptosis such as Bax and cytochrome c, caspase-3 and -8 in cisplatin-treated cells. The survival effect of NaHS was higher in HEI-OC1 cells cultured with 25 mM of glucose than in 5 mM of glucose. Interestingly, the antisurvival effect of H_2_S donors (NaHS or the slow donor GYY4137) was higher when cancer HepG2 and MCF7 cells were cultured with medium containing 25 mM of glucose rather than 5 mM of glucose [32]. In contrast with our results, minimal differences in survival were found in two non-tumoral cell lines [32]. Therefore, both survival and antisurvival effects might be cell line- and glucose-dependent.

It is worth noting that the dysregulation of H_2_S producing enzymes has been found in several cancer types, and H_2_S is involved in the regulation of many biological processes (proliferation, apoptosis, migration, autophagy, angiogenesis) that are important in the progression or the suppression of cancer. The effect of H_2_S appears to depend on the concentration, time and cell type (reviewed in [45]).

In HEI-OC1 cells, NaHS decreases ROS levels and increases the transcription of genes involved in the antioxidant response. Our results demonstrate that transcriptional levels of *Nrf2* increase after treatment with cisplatin, in agreement with Rybak and colleagues [46]. The transcription of several Nrf2 targets, but not that of *Nrf2* itself, increases after treatment with the combination of NaHS plus cisplatin for 24 h. We cannot exclude the possibility of an earlier NaHS-mediated increase in *Nrf2* transcription and activity, but the effect of NaHS on the Nrf2 target genes in HEI-OC1 cells could be due to an increase in sulfhydration and the degradation of Keap1, leading to nuclear translocation and activation of Nrf2 [47,48]. We have observed an increase in the transcription of *Hmox1*, *Sod2*, *Nqo1* and *Gclc*. These results suggest that the NaHS-mediated reduction in ROS could be due to the increase in the expression of enzymes catalyzing the destruction of ROS or the increase in the endogenous antioxidant glutathione. The glutamate-cysteine ligase is the first rate-limiting enzyme of glutathione synthesis. An increase in dismutation of superoxide ion leading to the production of H_2_O_2_ and O_2_ catalyzed by mitochondrial Sod2 as well as the action of the enzymes Hmox1 and Nqo1, which are considered to be crucial for the maintenance of the redox homeostasis of cells, could lead to a decrease in ROS [49,50]. In addition, Qiang et al. [51] have suggested that Nrf2 is able to inhibit ferroptosis via pSTAT3 by significantly increasing the levels of glutathione. STAT1 and STAT3 have opposing effects in the cochlea. STAT1 is pro-inflammatory and appears to mediate cisplatin-induced apoptosis [52] while STAT3 acts as a pro-survival molecule contributing to the resolution of inflammation [53]. Cisplatin reduces pSTAT3 expression in HEI-OC1 cells but cotreatment with NaHS for 24 h is not able to significantly increase it. Whether NaHS could affect STAT-3 activation at a different time point or with a different mechanism such as acetylation or methylation [54] requires further investigation. We have demonstrated that cisplatin induces an increase in the expression of pNFkB. This result is in agreement with So et al. [39], who demonstrated that the cisplatin-induced nuclear translocation of NFkB is associated with increased synthesis and release of pro-inflammatory cytokines, including TNFα and IL-6. The protective effect of NaHS was not associated with a decrease in the activation of NFkB in our experimental conditions, but it was associated with a decrease in aSMase activity. aSMase catalyzes the formation of ceramide from sphingomyelin and plays a central role in the cellular response to oxidative stress and in the control of cellular fate [55]. aSMase is involved in cisplatin-induced apoptosis and ferroptosis in several types of cells [36,37,56,57]. Cisplatin induces the secretion of TNFα and interleukin-1 which are known to stimulate the synthesis of secretory aSMase [58] and to stimulate caspase-8 and -3 [55]. aSMase activation has been associated with the degradation of glutathione peroxidase 4 [56,59], an enzyme that catalyzes the reduction of lipid hydroperoxides and thereby protects cells against oxidative damage. Novgodorov et al. [56] have found that aSMase activation is associated with a decrease in the level of glutathione. Therefore, it is possible that the NaHS-mediated increase in viability could be related, at least in part, to an increase in glutathione levels leading to a decrease in aSMase activity, less degradation of glutathione peroxidase 4 and less ferroptosis in HEI-OC1 cells, although this issue needs further investigation.

Since the effect of NaHS on the reduction of cisplatin-induced cytotoxicity is not specific for auditive cells, the local delivery of NaHS should be considered in order to protect the cochlea without decreasing the antitumoral effect of cisplatin. Studies in animal models have explored this possibility [60,61]. Much effort is being dedicated to optimize drug delivery through the intratympanic administration of hydrogels and nanoparticles. Intratympanic drug delivery has already been used in humans for intractable Menière’s disease [62], for idiopathic sudden hearing loss [63,64], and recently, for cisplatin-induced ototoxicity [65,66]. The last studies have demonstrated that intratympanic injection of thiosulfate is feasible and safe, but the number of patients was very small, and more work is necessary to establish whether and in which conditions (such as time of injection relative to cisplatin infusion) it is possible to obtain otoprotection.

Our results demonstrate that NaHS at a concentration higher than 250 μM exerts a protective effect on cisplatin-treated HEI-OC1 cells, that was not mimicked by erucin. These results suggest that H_2_S donors are interesting protective agents, but the time and dose concentration necessary to have a beneficial effect need to be revealed. In fact, erucin is a slow H_2_S donor [67] while sodium hydrosulfide hydrolysis produces a fast peak H_2_S release [68]. Therefore, it will be interesting to further investigate the effect of other H_2_S donors with different release mechanisms as well as hybrid donors that combine H_2_S-releasing capacity with other molecules able to induce cochlear cell protection such as glucocorticoids or lipoic acid providing antioxidant and anti-inflammatory action [13,69].

## 4. Materials and Methods

### 4.1. Materials

DCFH-DA, ethylenediaminetetraacetic acid (EDTA), fungizone, 3-(4,5-dimethylthiazol-2-yl)-2,5-diphenyltetrazolium bromide (MTT), phenylmethyl sulphonyl fluoride (PMSF), protease inhibitor cocktail sodium fluoride, sodium orthovanadate, β-glycerophosphate, sodium pyrophosphate, dimethylsulfoxide, were from Merck (Milan, Italy); cisplatin was from Teva (Assago, Italy), Dulbecco’s modified Eagle’s medium (DMEM), Ham′s F12, minimum essential medium, non-essential aminoacids, sodium pyruvate, fetal bovine serum (FBS), glutamine and trypsin were from Euroclone (Pero, Milan, Italy). N-Acetyl-Asp-Glu-Val-Asp-p-nitroaniline (AcDEVD-pNA), Ac-Ile-Glu-Thr-Asp-p-nitroaniline (Ac IETD-pNA), Ac-Leu-Glu-His-Asp-p-nitroaniline (Ac LEHD-pNA) and erucin were from Cayman (Ann Arbor, MI, USA). Amplex Red sphingomyelinase assay Kit, CellMask Orange and Hoechst 33342 were from Thermo Fisher scientific (Waltham, MA, USA). RNeasy Mini and QuantiTect Reverse Transcription Kits were from Qiagen (Hilden, Germany). Chemiluminescence Detection System and polyvinylidene fluoride (PVDF) membranes were from Millipore (Burlington, MA, USA); primary antibodies specific for NFkB (#8242), phospho-NFkB (Ser536) (#3033), horse radish peroxidase (HRP)-linked anti-mouse (#7076) and anti-rabbit IgG (#7074) were from Cell Signaling (Danvers, MA, USA), antibodies specific for STAT3 (#SC482), and phospho-STAT3 (Tyr705, #SC8059), were from Santa Cruz Biotechnology (Dallas, TX, USA); antibodies specific for Bcl2 (#ab194583) and Bax (ab182733) were from Abcam (Cambridge, UK). All other chemicals were of reagent grade. House Ear Institute-Organ of Corti 1 cells (HEI-OC1), U87 glioblastoma cells and SH-SY5Y were a gift from Prof. Federico Kalinec (UCLA, University of California Los Angeles, Los Angeles, CA, USA), Prof. Massimo dal Monte (University of Pisa, Pisa, Italy) and Prof. Annarosa Arcangeli (University of Florence, Florence, Italy), respectively.

### 4.2. Cell Culture

HEI-OC1 cells were maintained in high-glucose DMEM containing 1 mM pyruvate, supplemented with 10% FBS and 2.5 μg mL^−1^ amphotericin B at 33 °C in a humidified incubator with 10% CO_2_ as described [20]. U87 glioblastoma cells cultured at 37 °C in 5% CO_2_ in high-glucose DMEM supplemented with 10% FBS, 100 U/mL penicillin and 100 µg/mL streptomycin. SH-SY-5Y neuroblastoma cells were grown in Ham′s F12: Eagle’s minimum essential medium (1:1), 10% FBS, 100 U/mL penicillin, 100 µg/mL streptomycin and 2.5 μg/mL amphotericin B at 37 °C in 5% CO_2_.

### 4.3. Preparation of Lisosan G, Erucin, and Taurisolo^®^

Lisosan G was obtained by fermenting and drying whole wheat flour from *Triticum aestivum* grains as described in [21,31]. Briefly, starter cultures typically consist of a mix of *Lactobacillus* and natural yeast strains in a ratio of about 100:1 (Natural Sourdough). Once the product was fermented, it was dried using a vacuum pump at 20–25 °C temperature and 2 bar pressure until reaching 12% humidity (48–60 h for 100 kg material). For the treatments, Lisosan G was dissolved in distilled water, sterile-filtered, and kept at 4 °C in the dark until use. Erucin was dissolved in dimethyl sulfoxide and this solution (10^−2^ M) was freshly diluted in the appropriate culture medium. Taurisolo^®^ was obtained from Aglianico cultivar grape as described in [22].

### 4.4. Viability Experiments

Five thousand HEI-OC1 cells were seeded in 96-well plates, containing 100 µL of medium. The following day, cells were treated in the presence or absence of different concentrations of Lisosan G, Taurisolo^®^, erucin or NaHS and 5 μM or 10 μM cisplatin. After 24 h or 48 h, cell viability was measured as MTT reduction. Briefly, 0.5 mg/mL MTT in phosphate-buffered saline was added to each well; cells were incubated at 33 °C in a humidified 10% CO_2_/90% air atmosphere for 60 min, the reaction was stopped by replacing the MTT solution with 100 µL dimethylsulfoxide, and the formazan salts were dissolved by gentle shaking for about 5 min at room temperature and quantified spectrophotometrically by reading the absorbance at 596 nm in an automatic ultra-microplate reader EL 808 Bio-Tek Instruments. Inc. (Winooski, VT, USA). Values were normalized considering the average absorbance of the control sample. Each experiment was performed in octuplicate and repeated at least two times.

### 4.5. Flow Cytometry: Annexin-V Assay

HEI-OC1 cells (1.7 × 10^5^ cells/well) were plated in 6-well dishes and, the following day, they were treated in the presence or absence of 10 µM cisplatin and 0.5 mM NaHS for 24 h. Apoptosis was evaluated using the FITC Annexin V/Dead Cell Apoptosis Kit (Invitrogen, Thermo Fisher Scientific, Waltham, MA, USA). The cells were harvested by trypsinitation, washed with PBS and both the harvested cells and the wash containing detached cells were pelleted by centrifugation. Cells were resuspended in annexin-binding buffer and FITC annexin V and propidium iodide were added. Results were analyzed by flow cytometry, which was performed using the Attune NxT Acoustic Focusing Cytometer (Invitrogen, Thermo Fisher Scientific). Gating was set at 50,000 cells/sample.

### 4.6. Cell Extract Preparation

Three hundred thousand cells were seeded in 60 mm diameter plates and incubated both in the presence and in the absence of 10 µM cisplatin and/or NaHS. After 24 h or 48 h incubation, cells were washed with 1 mL cold PBS supplemented with the protease inhibitor cocktail, 1 mM PMSF, 1 mM sodium orthovanadate, 5 mM sodium pyrophosphate, 20 mM β-glycerophosphate. The wash was then removed, and the cells were scraped off and collected. The plates were then washed with 1 mL cold PBS containing protease inhibitors and the remaining cells were collected and added to the previous ones, then centrifuged at 700× *g* for 5 min at 4 °C. Supernatants were discarded and 30 μL of lysis buffer containing 150 mM NaCl, 50 mM NaF, 0.5 mM EDTA pH 8.0, 1% Triton X-100, 1 mM dithiothreitol, 1 mM PMSF, 1 mM sodium orthovanadate, 5 mM sodium pyrophosphate and 20 mM β-glycerophosphate in 25 mM Tris-Cl pH 7.4 was added. Vials with cells and lysis buffer were then strongly shaken for 1 min and kept on ice for 30 min prior to centrifugation at 10,000× *g* for 15 min at 4 °C. The supernatants were collected, and the concentration of protein extracts was determined by the Bradford method. Aliquots were kept at −80 °C and used to measure caspase activity or for Western blotting.

### 4.7. Western Blot

Protein samples (30 μg each lane) were resolved by 12% SDS-PAGE using TGX Stain-free^TM^ FastCast^TM^ acrylamide (Bio-Rad Laboratories, Hercules, CA, USA) at 200 V for 45 min. Proteins were transferred onto PVDF membranes using the Trans Blot Turbo Transfer system (Bio-Rad). Membranes were blocked with TBSTa (50 mM Tris-HCl pH 7.5 supplemented with 150 mM NaCl, 0.1% (*v/v*) Tween-20 and 5% (*w/v*) skim milk powder) or TBSTb (50 mM Tris-HCl pH 7.5 supplemented with 150 mM NaCl, 0.1% (*v/v*) Tween-20 and 5% (*w/v*) bovine serum albumin) for 1 h. Membranes were incubated with primary antibody overnight at 4 °C, then secondary antibody was added and kept for 1 h at room temperature before visualizing the chemiluminescence of protein bands using the ChemiDoc imaging system (Bio-Rad). Primary antibodies specific for STAT3 (1:200), NFkB (1:1000), Bax (1:500), Bcl2 (1:500) and cytochrome c (1:250) in TBSTa, pSTAT3 (1:200), pNFkB (1:1000) in TBSTb and HRP-linked secondary antibodies anti-mouse (1:5000) and anti-rabbit IgG (1:1000) in TBSTa were used. The relative abundance of proteins was determined using Bio Rad Image Lab version 6 software using the Stain free (Bio-Rad) technology as a loading control [70].

### 4.8. Caspase Activity

Determination of the activity of caspase-3, caspase-8 and caspase-9 was carried out in a 96-well plate in a total volume of 100 µL of 50 mM Tris-HCl pH, 10 mM dithiothreitol using 150 µg protein/200 µM DEVD-pNA, 100 µg protein/100 µM Ac-IETD-pNA and 100 µg protein/200 µM Ac-LEHD-pNA, respectively. Extracts were incubated for 4 h at 37 °C in the presence of the corresponding tetrapeptide conjugated to paranitroaniline (pNA) and the released pNA was measured in a spectrophotometer at 405 nm every 60 min.

### 4.9. Measurement of Intracellular ROS Production

The intracellular ROS level was measured by using DCFH-DA, which is converted to fluorescent 2′, 7′-dichlorofluorescein in the presence of an oxidant. For the assay, HEI-OC1 cells (1.7 × 10^5^ cells/well) were plated in 6-well dishes and, the following day, they were treated in the presence or absence of 10 µM cisplatin and 500 µM NaHS for 24 h. Cells were washed and incubated in the dark for 30 min at 37 °C with 10 µM DCFH-DA. Cells were tripsinized and resuspended in 500 µL DEMEM without FBS and phenol red and 1 µL of propidium iodide was added (final concentration 1 µg/mL). Fluorescence was analyzed by using the Attune NxT Acoustic Focusing Cytometer (Invitrogen, Thermo Fisher Scientific) at an excitation wavelength of 495 nm and an emission wavelength of 530 nm (FL-1) with gating at 50,000 cells/sample.

### 4.10. High-Content Confocal Imaging

Five thousand cells were seeded in 96 CellCarrier Ultra plates (PerkinElmer, Hamburg, Germany), then treated as described above. Cells were incubated for 30 min with 10 μM DCFDA, 1 μg/mL CellMask Orange (to stain cellular membranes) and 1 μg/mL Hoechst 33342 (to stain nuclei). Images were acquired using the Operetta CLS high-content imaging device (PerkinElmer, Hamburg, Germany) using a 40× water objective and analyzed with Harmony 4.6 software (PerkinElmer, Hamburg, Germany). Nine fields were acquired per well, with 8 experimental replicas.

### 4.11. Reverse Transcription Quantitative Real-Time PCR

Quantitative real-time PCR (qPCR) was used to determine the expression of oxidative stress markers, including *Nrf2*, *Sod2*, *Hmox1*, *Nqo1* and *Gclc*. Three hundred thousand cells were seeded in 60 mm diameter plates and incubated both in the presence and in the absence of 10 µM cisplatin and/or NaHS for 24 h. Then, the cells were washed twice with 1 mL cold PBS. Total RNA was extracted, purified and resuspended in RNase-free water using an RNeasy Mini Kit. After spectrophotometric quantification, 1 μg of total RNA was used to generate the first-strand cDNA using a QuantiTect Reverse Transcription Kit (Qiagen). qPCR was performed using SsoAdvanced Universal SYBR Green Supermix on a CFX Connect Real-Time PCR Detection System and software CFX manager v3.1 (Bio-Rad Laboratories). qPCR primer sets were chosen to hybridize unique regions of the appropriate gene sequence [29].

The expression of *Rpl13a*, a constitutively expressed gene encoding for ribosomal protein L13a, was used as an endogenous control and housekeeping gene for normalization. Samples were compared using the relative threshold cycle (Ct method). The increase or decrease (fold change) was determined relative to the experimental control group after normalization to *Rpl13a*.

### 4.12. Acid Sphingomyelinase Activity

Nine hundred thousand cells were seeded in 10 mm diameter plates and incubated in the presence or absence of 30 μM cisplatin and 2 mM NaHS for 24 h. Cells were suspended in 0.1% Nonidet P-40 detergent in phosphate-buffered saline containing the protease inhibitor cocktail, sonicated for 30 s on ice, kept on ice for 30 min, and centrifuged at 10,000× *g* for 15 min. The supernatants were then used for the aSMase assay. The enzyme activity was assayed in 100 µg proteins/100 µL reaction buffer pH 5.0, using an Amplex Red Sphingomyelinase assay kit, according to the manufacturer’s instructions. The fluorescence was measured with a FLUOstar Optima fluorimeter (BMG Labtech, Ortenberg, Germany) using a filter set with a 544 nm excitation and 590 nm emission.

### 4.13. Statistical Analysis

Data are presented as the mean ± SEM of the respective n values (Prism 9; GraphPad software, San Diego, CA, USA). The results are compared using ANOVA followed by Tukey’s multiple comparisons test. A value of *p* < 0.05 was considered significant.

## Data Availability

The data presented in this study are available on request from the corresponding author.

## Supplementary Materials

The following supporting information can be downloaded at: https://www.mdpi.com/article/10.3390/ijms242417416/s1.
