# Supplementary material for: Effects of Nutraceuticals on Cisplatin-Induced Cytotoxicity in HEI-OC1 Cells
Source: Int J Mol Sci. 2023 Dec 12;24(24):17416. doi: 10.3390/ijms242417416 (PMC10743635; doi:10.3390/ijms242417416)
Supplement: Supplementary file 1 [file ijms-24-17416-s001.zip › ijms-2736787-supplementary.pdf]

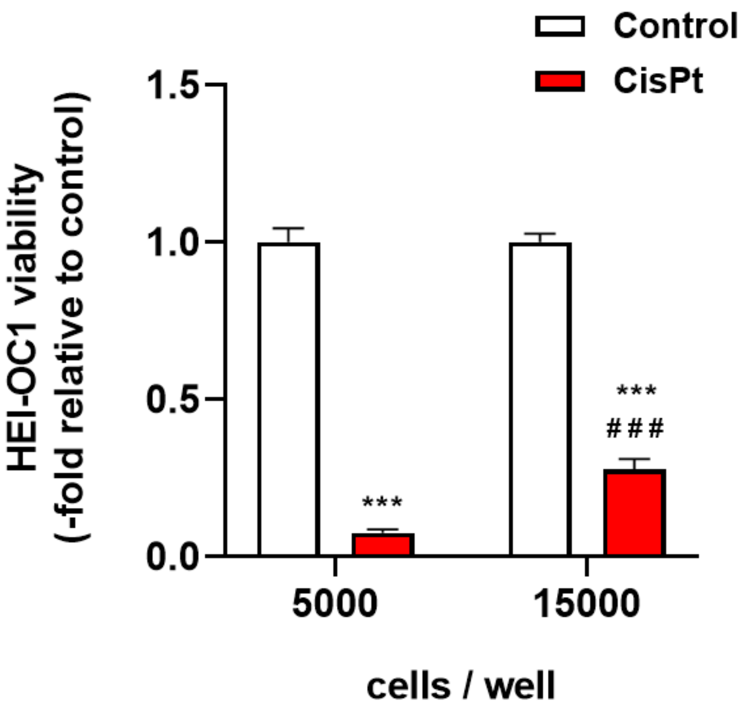

Figure S1. Effect of cell density on the viability of HEI-OC1 cells. HEI-OC1 cells were seeded in 96-well plates at the density indicated, incubated for 48 h with 10 μM cisplatin (CisPt) and viability was measured as described in Materials and Methods. Data were normalized to the absorbance of cells incubated in the absence of cisplatin (control) and are expressed as mean ± SEM (n = 2 performed in octuplicate). The reduction of viability induced by cisplatin was inversely related to cell density. Significance: *** $p < 0.001$ vs control; ### $p < 0.001$ vs cisplatin 5000 cells/well.

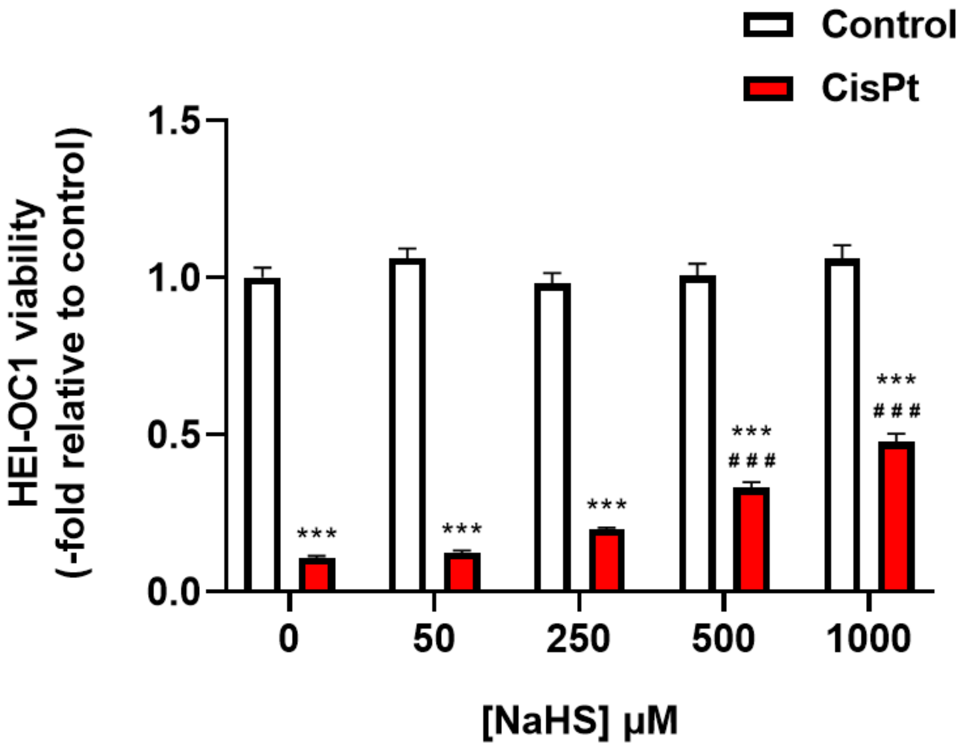

Figure S2. Five thousand cells were seeded in 96-well plates and incubated in the presence or the absence of different concentrations of NaHS and 10 μM cisplatin for 48 h in DMEM containing 1mM pyruvate (as in figure 2D) but only 5 mM glucose). Data were normalized to control cells (grown in the absence of cisplatin and NaHS) and are expressed as mean ± SEM (n = 2 performed in octuplicate). Significance: *** $p < 0.001$ vs control; ### $p < 0.001$ vs cisplatin. CisPt, cisplatin; NaHS, sodium hydrosulfide.

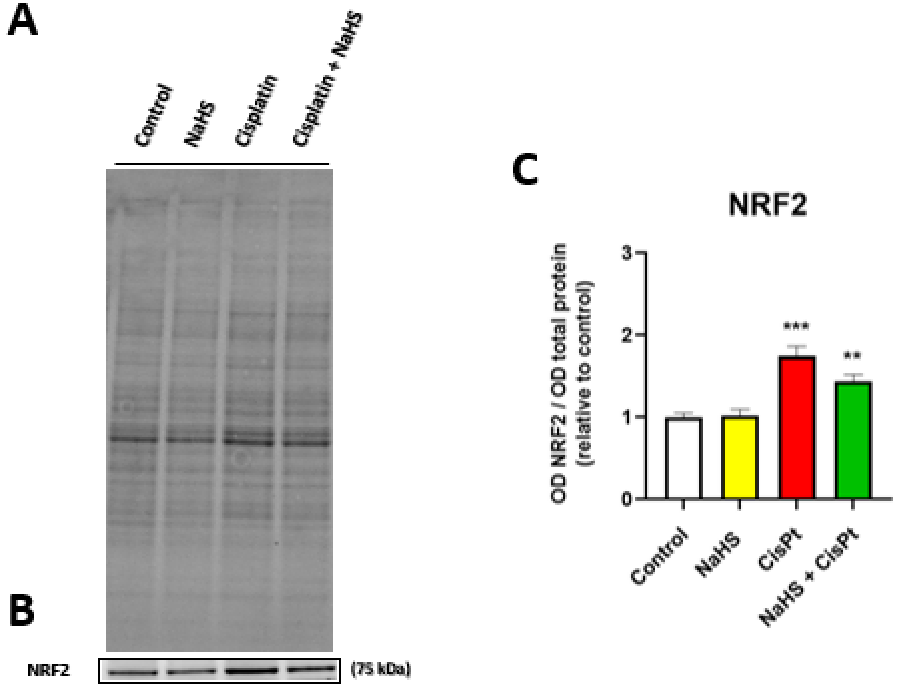

Figure S3. Effect of cisplatin and NaHS on Nrf2 expression in HEI-OC1 cells. HEI-OC1 cells were treated for 24 h in the presence or absence of 10 μM cisplatin and 500 μM NaHS. (**A**) Representative example of total transferred proteins. (**B**) Representative example of Western blots. (**C**) Quantification of the expression of Nrf2 ($n = 5$). Data have been normalized to the total quantity of proteins and to the control and are expressed as mean ± SEM. Statistical significance: ** $p < 0.01$, *** $p < 0.001$ vs control; vs cisplatin. CisPt, cisplatin; NaHS, sodium hydrosulfide; NRF2, nuclear factor erythroid 2-related factor.

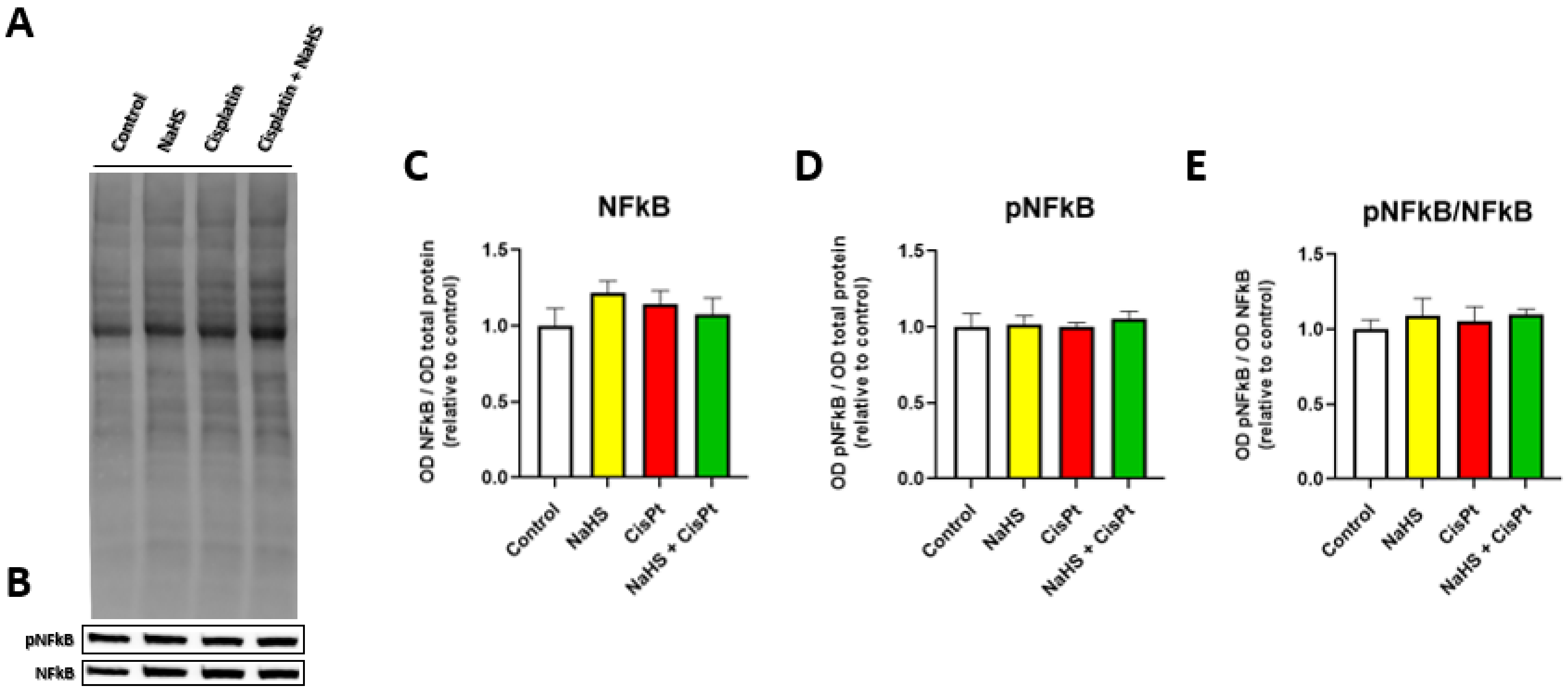

Figure S4. Effect of NaHS and cisplatin on NFkB and pNFkB expression in HEI-OC1 cells. HEI-OC1 cells were treated for 6 h in the presence or absence of 10 μM cisplatin and 500 μM NaHS. (**A**) Representative example of total transferred proteins. (**B**) Representative example of Western blots. (**C**–**E**) Quantification of the expression of NFkB, pNFkB, and the ratio pNFkB/NFkB (*n* = 4). Data have been normalized to the total quantity of proteins and to the control and are expressed as mean ± SEM. CisPt, cisplatin; NaHS, sodium hydrosulfide; NFkB, nuclear factor kappa B; pNFkb, phosphorylated NFkB.
